# Utility of Seizure Pattern and Related Clinical Features in the Diagnosis of Neurometabolic Disorders

**Published:** 2020

**Authors:** Narjes JAFARI, Asieh MOSALLANEJAD, Asieh GHOBADIFAR, Parvaneh KARIMZADEH, Robabeh Ghodssi GHASSEMABADI, Mohammadmehdi NASEHI, Marjan SHAKIBA, Shahrzad TABATABAEE

**Affiliations:** 1 Department of Pediatric Neurology, Pediatric Neurology Research Center, Research Institute for Children’s Health, Shahid Beheshti University of Medical Sciences, Tehran, Iran.; 2 Pediatric endocrinology and metabolism Department ,Mofid Children's Hospital, Shahid Beheshti university of medical science , Tehran, Iran.; 3 Pediatric Neurology Department, Mofid Children’s Hospital, Faculty of Medicine, ShahidBeheshti University of Medical Sciences, Tehran, Iran.; 4 Imam Hosein Medical Center, Shahid Beheshti University of Medical Sciences, Tehran, Iran.; 5 Department of Biostatistics, Faculty of Medical Sciences, Tarbiat Modares University, Tehran, Iran.

**Keywords:** Seizure, Children, Neurometabolic, Disorders

## Abstract

**Objectives:**

The current study aimed at identifying the role of seizure types and related clinical features in differentiation between neurometabolic disorders and other causes of seizure.

**Materials & Methods:**

The current cross sectional study was conducted at two referral children hospitals in Tehran, Iran, from 2011 to 2018. The study population included 120 patients presenting with seizure due to neurometabolic disorders and 120 cases due to other causes. The types of seizure and related clinical findings were assessed in both groups.

**Results:**

There was a significant difference in the frequency of seizure types in the two groups. Tonic and myoclonic seizures as well as infantile spasm were observed more commonly in the patients with neurometabolic disorders, while atonic, partial and generalized tonic-clonic seizures were more common in the control group. In addition, frequency of refractory seizure, age at onset of seizure, and pattern of involvement in brain imaging were helpful for differentiation.

**Conclusion:**

The pattern of seizure and related findings varied in patients with metabolic disorders, and was helpful for diagnosis. Thus, these factors can contribute to early diagnosis and treatment.

## INTRODUCTION

Inborn error of metabolism (IEM) is a group of disorders in which genetic abnormalities at the cellular level result in the lack or dysfunction of specific enzymes, cofactors, or transporters required for fatty acid, amino acid, or carbohydrate metabolism (1). Some IEM disorders affecting the nervous system are considered as neurometabolic disorders. Brain is susceptible to damage in such situations (2). Seizure is a common manifestation in neurometabolic disorders and may be considered as a presenting symptom. Epilepsy is not rare in the general population (seizures occur in one per 1000 live births) (3). Seizure is a common symptom of metabolic diseases, reported in about 200 different IEM disorders (3); however, there are limited data on the certain seizure patterns in patients with metabolic disorders (1, 2, 4-6). Therefore, some specific features are reported in seizures along with neurometabolic disorders [7].

Early onset seizures and refractory seizures accompanied by developmental disabilities are commonly observed in IEM disorders [8].

Regarding seizure, clinical findings are often nonspecific (9, 10), and high index of suspicion is needed to consider neurometabolic disturbances. Sometimes, specific features of seizure and other clinical-related findings may indicate metabolic disorders. Early detection of disturbance and appropriate management of pathological conditions can prevent comorbidity and improve the quality of life. Therefore, the current case-control study aimed at assessing the seizure pattern and its related findings in patients with neurometabolic disorders in order to report common types of seizure and other clinical manifestations to differentiate between neurometabolic disorders and other causes of seizure.

## Materials & Methods

 The current case-control study aimed at comparing the characteristics of seizure and other valuable findings in patients with neurometabolic disorders and those of a control group. The study was performed in the pediatric neurology wards of Mofid Children's and Imam Hossein hospitals, two tertiary care referral centers, from 2011 to 2018, in Tehran, Iran. A total of 213 patients diagnosed with neurometabolic disorders were enrolled. Diagnosis was made according to clinical manifestations, physical and neurologic examinations, neuro-imaging findings, and finally with enzymatic, biochemistry, or genetic-based studies. Videlicet, the diagnosis of metabolic disorders, was based on biochemical tests (i e, serum acylcarnitine profile, and organic acidurias in organic acidemia, serum amino acids in aminoacidopathy by high-performance liquid chromatography, very long-chain fatty acid in peroxisomal disorders, and enzyme assay in storage diseases). In some challenging cases, the right diagnosis was confirmed using genetic tests. 

Of the 213 studied subjects, 55% (n=120) had seizure disorders. These patients were considered as the case group. Neurometabolic disorders in the case group included organic academia, aminoacidopathy, and urea cycle disorders, storage disease, leukodystrophy, fatty acid oxidation disorders, and peroxisomal diseases. The age range of the patients was 1 to 196 months.

The control group included 120 children within the age range of 1 to 196 months referred to the same hospitals with seizure for other reasons (i e, genetic disorders, acquired disorders such as brain tumors, inflammatory diseases, electrolyte imbalances, trauma, withdrawal syndromes, hemorrhage, hypoxic ischemic encephalopathy, prematurity insult, brain structural disorders, and specific epileptic syndromes).

The exclusion criteria were neonatal period, febrile convulsion, and incomplete data. The clinical findings including demographic information, age at onset of seizure, family history of seizure, developmental delay or regression, consanguinity, type of seizure (tonic or myoclonic seizure, infantile spasm, and atonic, partial, generalized tonic-clonic (GTC) or absence seizure), refractory seizure, and specific pattern of electroencephalography (EEG), as well as magnetic resonance imaging (MRI) results were recorded in a form. Other findings such as eye abnormalities, skin and hair problems, and hearing impairments were also recorded.

Finally, the characteristics were compared between the two groups. The study was approved by the Ethics Committee of Shahid Beheshti University of Medical Sciences, Tehran, Iran. All patients were included in the current study with parental informed consent.

Statistical analysis

Continuous data were expressed as mean ± standard deviation and categorical data as frequency and percentage. In order to compare the characteristics and outcomes between the two groups, independent samples t-test, the Mann Whitney and Chi-squared tests were applied and the exact P-values were calculated whenever needed. The significance level was set at 0.05 and SPSS version 21 was used for data analysis. 

## Results

Of the visited 213 patients with neurometabolic disorders, 55% (n=120) had seizure disorder (case group). Pattern of seizure and related clinical and paraclinic findings in the case group were compared with those of the 120 cases with seizure secondary to other causes.

Mean age of the patients at the time of evaluation was significantly different between the case and control groups (39.86± 47.43 vs. 73.76± 51.18 months; P=0.001). There were 56 female and 64 male patients in the case group and 60 female and 60 male patients in the control group, indicating an insignificant difference between the groups in this regard (P=0.698). Age at the onset of seizures was significantly lower in the case group than the control group (13.20 ± 21.40 vs. 40.90 ± 36.50 months; P <0.001), data are shown in Table 1.

Distribution of the types of seizure was significantly different between the two groups (P <0.001); tonic and myoclonic, infantile spasm, and mixed-type seizures were more common in the case group, while atonic, absence, partial, and GTC seizures were more frequently observed in the control group, data are shown in Table 2. Overall, 80.6% of tonic seizures were observed in the case group, while just fewer than 20% of them occurred in the control group. Two-thirds of myoclonic seizures happened in patients with metabolic disorders, and a third in the control group. In addition, 57.1% of cases with epileptic spasm were observed in the neurometabolic patients . 62.5% of mixed-type seizures were reported in the case group. In contrast, almost 89% of partial seizures were detected in the control group, while only 11% of them happened in the case group. 

Similarly, around 93% of atonic seizures were detected in the control group, and all patients with absence seizure were in the control group. Also , 79.7% of GTC seizures were observed in the control group, and refractory seizure was observed in 14.5% of the case and 4.2% of the control groups. There was no significant difference between the case and control groups in terms of the family history of seizure (34 (28.3%) in cases vs. 31 (25.8%) in controls; P=0.663).

**Table1:** Demographic Characteristics of the Study Groups

	Group		
Variable	Patients With Neuro-metabolic Disorders (N=120)	Control (N=120)	
	Mean ± SD	Mean ± SD	P-value
Age (mn)	39.86 ± 47.43	73.76 ± 51.18	<0.001
Age at onset of seizures (mn)	13.20 ± 21.40	40.90 ± 36.50	<0.001
	N (%)	N (%)	
Gender Male	56 (46.67)	60 (50)	0.698
Female	64 (53.33)	60 (50)	

**Table 2:** The Frequency of Seizure Types in the Case and Control Groups

	Group		Total (N=240)
Seizure Type	Control (N=120)	Case (N=120)	
Tonic	6 (5)	35 (29.2)	41 (17.1)
Myoclonic	3 (2.5)	10 (8.3)	13 (5.4)
Atonic	13 (10.8)	2 (1.7)	15 (6.3)
Partial	24 (20)	5 (4.2)	29 (12.1)
Generalized tonic-clonic	55 (45.8)	27 (22.5)	82 (34.2)
Absence	7 (5.8)	1 (0.8)	8 (3.3)
IS	3 (2.5)	8 (6.7)	11 (4.6)
Others	3 (2.5)	19 (15.8)	22 (9.2)
Mixed	6 (5)	13 (10.8)	19 (7.9)

Data are expressed as frequency (percent).

IS: Infantile spasm

Parental consanguinity was significantly higher in the case group subjects compared with the control group ones (82.5% vs. 22.5%; P <0.001), Table 3. Overall, 95% of patients in the case group and 5% in the control group had abnormal neurologic status (P <0.001). For example, ocular problems were observed in 31.7% of the cases and none of the controls (P <0.001. Dysmorphic feature was noted in 10.8% of the subjects in the case group and none of the control group patients (P <0.001). Skin and hair problems were observed in a quarter of the patients in the case group and none of the control group subjects (P <0.001). Also, hearing impairment was detected in 9.1% of the case group patients (5.8% with hyper acusis and 3.3% with hearing loss) and none of the control group subjects (P=0.009).

Growth velocity of head circumference was considerably different between the case and control groups (60% vs. 5.8%; P <0.001). Accordingly, microcephaly appeared in 27.5% of case group subjects and 4.2% of the control patients, in the same vein, macrocephaly was observed in 23.3% of cases and 1.7% of the controls. 

In patients with neurometabolic disorders, 92.5% had abnormal developmental status, whilst only 22.5% of the patients in the control group had developmental disability. In addition, of these patients, in the case group 56.7% appeared with developmental delay, 20.8% with delay plus regression, and 15% with regression, while in the control group only developmental delay was observed without regression. Developmental regression was observed in 35.8% of the case group subjects, which was not detected in any of the patients in the control group (P=0.0002). 

**Figure1:**
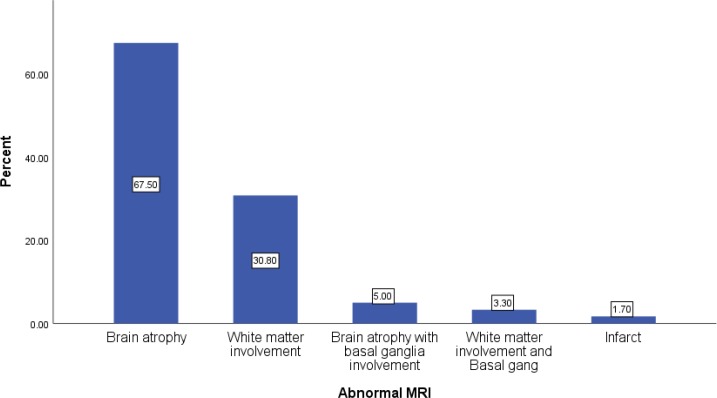
Abnormal MRI Distribution in the Case Group

Brain MRI Valuable findings were observed in 62.5% of the case vs 21.7% of the ones in the control groups, which were significantly different (P=0.0001). In patients with metabolic disorders, white matter involvement was seen in 23 and brain atrophy in 50 cases. In six patients brain atrophy was associated with basal ganglia and two cases had white matter involvement along with basal ganglia anomaly. Infarct was observed in two patients, too.(Figure 1).

## Discussion

Despite the rarity of the IEM disorders, they are important and fairly common causes of seizure in children. When patients present with seizure, it is challenging to diagnose neurometabolic disorders (1). In patients with metabolic diseases, severe morbidity and mortality can be prevented by early detection and proper management strategies. However, diagnosis in such patients can be problematic and expensive, thus, diagnostic keys are important in this process. The current study evaluated 213 patients with metabolic disorders, 55% (n=120) of whom had seizure disorders. Patients with seizure due to neurometabolic disorders were assessed and compared with 120 patients with seizure, secondary to other causes, and then the key points were specified to metabolic disorders. A clear difference was observed in the type of seizures between the two groups; tonic and myoclonic seizures, infantile spasm, and mixed seizures were considerably more common in patients with neurometabolic disorders, whereas partial, atonic, absence, and GTC seizures were observed more frequently in the control group.

Previous studies revealed that seizure is a common presentation of neurometabolic disorders to such an extent that 40%-90% of patients with such problems experience seizure (2, 11). Several studies indicated that some types of seizure are more common in neurometabolic disorders; e g, infantile spasm and myoclonic seizure (2, 12). In the current study, seizure started earlier in patients with neurometabolic disorders than the subjects in the control group (mean age: 39.86 vs. 73.76 months), which was in accordance with previous studies that reported ≤12 months old similar to the age at onset of seizure in 50%-60% of patients with neurometabolic disorders (1, 12). Age is an important factor to determine the type of metabolic disorder. In some of these disorders, seizure presents in lower ages, and in some others in higher ages; for example, in non-ketotic hyperglycinemia or pyridoxine deficiency, seizures start from the neonatal period (3); besides, the type of seizure also depends on age. Due to incomplete synchronization in infancy, GTC is rarely observed, and absence seizures are usually not observed until the age of two years. On the other hand, infantile spasm is observed during the neonatal period (13); thus, probably due to the occurrence of metabolic diseases at earlier ages, certain types of seizure are more common in such patients.

At baseline, 14.5% of patients with metabolic disorders showed refractory seizure symptoms versus 4.2% in the control group. It was clear that some of these patients needed special treatments for correction of metabolic abnormalities, and their seizure could not be controlled solely with antiepileptic drugs (14).

Overall, 95% of the patients with neurometabolic disorders had abnormal physical manifestations, while the rate was only 5% in the control group. These abnormalities included vision and ocular problems in one-third of the patients in the case group, dysmorphic face in 10.8%, and hair and skin problems in 25% of the subjects in the same group of patients, whilst none of these problems were observed in the control group. In addition, abnormal head circumference was observed in 60% of the patients in the case group versus 5.8% of the controls, hearing impairment was observed in 9.1% of the patients in the case group, but none in the controls. Also, developmental disability was observed in 92.5% of the case group subjects, which was 22% in those of the control group, additionally, 35.8% of the patients with neurometabolic disorders revealed developmental regression, which was not observed in the control group. The current study findings were in line with the results of previous studies; they reported multi-systemic presentations in patients with neurometabolic disorders including macrocephaly, microcephaly, dysmorphic features, skin lesion, foul odor, eye abnormalities, hearing impairment, and neuropathy (14, 15).

The presence of some of these findings helped to diagnose some specific metabolic disorders; for example, cherry-red spot was common in most patients with GM2 gangliosidosis or skin lesion was a diagnostic key for biotinidase deficiency (16, 17). Developmental regression is an important diagnostic key for metabolic diseases when patients present with seizure, which is less expected concomitant with seizures due to other causes (10).

In the current study results, the percentage of parental consanguinity in patients with neurometabolic disorders was significantly higher than that of controls (82.5% vs. 22.5%), which was in accordance with the findings of Sarar and Masry that reported the frequency of neurometabolic disorders in 75% and 77.1% of patients with parental consanguinity, respectively (1, 4); it can be explained by the fact that neurometabolic disorders are among autosomal recessive disorders.

 Family history of seizure disorders was positive in approximately one-third of the current study patients in both groups, and there was no significant difference between them in this respect. Previous studies revealed positive family history in 22%-33% of patients with neurometabolic disorders (1, 12), which was in accordance with the current study results, but those studies did not have a control group for comparison. Nevertheless, according to the current study design, history of seizure (not seizure plus neurometabolic disorder) was considered in the patients; thus, no significant relationship was observed in this regard, although it might exist. 

In the current study, electroencephalographic features did not help for the diagnosis of IEM. In other assessments, the distinctive EEG pattern was not proved for any IEM disorders, either (4).

Brain MRI in 62.5% of the patients with neurometabolic disorders elicited valuable diagnostic findings, while only 21.7% of the patients in the control group had abnormal brain MRI. In most of such patients, imaging was a great help to determine the type of metabolic disease; for example, MRI in all patients with Canavan disease showed subcortical, periventricular white matter leukodystrophy (18) or leukodystrophic changes in the posterior region with marginal post-contrast enhancement that was specific to adrenoleukodystrophy (ALD) (19). Additionally, Shamima reported abnormal brain imaging in two-thirds of patients and noted that it can be the diagnostic key for some types of neurometabolic disorders (3). Moreover, Zimmerman in his study underscored MRI and MR spectroscopy as valuable methods to evaluate children suspected of neurometabolic disorders (17). 

Suvasini et al., noted that the points for diagnosis of neurometabolic disorders in patients with epilepsy contained: positive family history, consanguinity of marriage, developmental disability, some other neurological manifestation such as hypotonia, abnormal ophthalmological exam, movement disorders, and some related systemic findings such as organomegaly, failure to thrive, cardiomegaly, etc. (20).

Ultimately, when managing a patient with epilepsy, neurometabolic disorders should be suspected if the patient meets one or more of the following criteria:

1. Certain types of seizures including tonic, myoclonic, infantile spasm, or mixed-type seizure

2. Seizures that initiated at a young age and resistant to common anti-epileptic drugs

3. Abnormal findings, in favor of metabolic disorders, such as macrocephaly or microcephaly, organomegaly, skin and hair problems, eye problems, hearing impairment, and developmental delay, especially regression 

4. The offspring of a consanguineous marriage

5. Positive family history of the same disease

6. Brain MRI indicating a diagnostic finding such as leukodystrophy or basal ganglia involvement

If patients with seizure do not meet the mentioned criteria, assessment for metabolic disorders such as the underlying disease cannot be helpful.

**Table2:** Comparison of Related Clinical Findings in the Case and Control Groups

	Group		
Variable	Patients With Neuro-metabolic Disorders (N=120)	Control (N=120)	
	Mean ± SD	Mean ± SD	P-value
	N (%)	N (%)	
Refractory seizure	18 (14.5)	5 (4.2)	<0.001
Abnormal head circumference	72 (60)	7 (5.8)	<0.001
Ocular problems	38 (31.7)	0 (0)	<0.001
Skin/hair problems	30 (25)	0 (0)	<0.001
Hearing impairment	11 (9.1)	0 (0)	<0.001
Hyperacusis	7 (5.8)		
Hearing loss	4 (3.3)		
Developmental delay	111 (92.5)	27 (22.5)	<0.001
Developmental regression	43 (35.8)	0 (0)	<0.001
Parental consanguinity	99 (82.5)	27 (22.5)	<0.001
Positive family history	34 (28.3)	31 (30.8)	0.771
Abnormal EEG	98 (81.6)	85 (70.8)	0.069
Abnormal brain MRI	75 (62.5)	26 (21.7)	<0.001

EEG: electroencephalography; MRI: magnetic resonance imaging

**In Conclusion,** In patients with seizure, some related findings sush as specific patterns of seizure, onset of seizure at a low age, refractory seizures, developmental disability- specially developmental regression- parental consanguinity, and key points in exams such as hearing or visual system abnormality, hair and skin abnormalities, and abnormal head circumference along with specific pattern of brain involvement in MRI are valuable keys to identify metabolic diseases.
